# Detection of Glutamate Alterations in the Human Brain Using ^1^H-MRS: Comparison of STEAM and sLASER at 7 T

**DOI:** 10.3389/fpsyt.2017.00060

**Published:** 2017-04-21

**Authors:** Anouk Marsman, Vincent O. Boer, Peter R. Luijten, Hilleke E. Hulshoff Pol, Dennis W. J. Klomp, René C. W. Mandl

**Affiliations:** ^1^Psychiatry, Brain Center Rudolf Magnus, University Medical Center Utrecht, Utrecht, Netherlands; ^2^Radiology, Brain Center Rudolf Magnus, University Medical Center Utrecht, Utrecht, Netherlands

**Keywords:** glutamate, magnetic resonance spectroscopy, reproducibility, brain, 7 T

## Abstract

**Purpose:**

To assess reproducibility of glutamate measurement in the human brain by two short echo time (TE) ^1^H-MRS sequences [stimulated echo acquisition mode (STEAM) and semi-localized by adiabatic selective refocusing (sLASER)] at 7 T. Reliable assessment of glutamate is important when studying a variety of neurological and neuropsychiatric disorders. At 7 T, the glutamate signal can be separated from the glutamine signal and hence more accurately measured as compared to lower field strengths. A sLASER sequence has been developed for 7 T, using field focusing at short TE, resulting in twice as much signal as can be obtained using STEAM and improved localization accuracy due to a decreased chemical shift artifact.

**Materials and methods:**

Eight subjects were scanned twice using both STEAM and sLASER. Data were acquired from the frontal and occipital brain region. Subsequently, intraclass correlations were computed for the estimated metabolite concentrations.

**Results:**

sLASER has higher ICC’s for glutamate concentration as compared to STEAM in both the frontal and occipital VOI, which is probably due to the higher sensitivity and localization accuracy.

**Conclusion:**

We conclude that sLASER ^1^H-MRS at 7 T is a reliable method to obtain reproducible measures of glutamate levels in the human brain at such high accuracy that individual variability, even between age-matched subjects, is measured.

## Introduction

**Figure 1:**
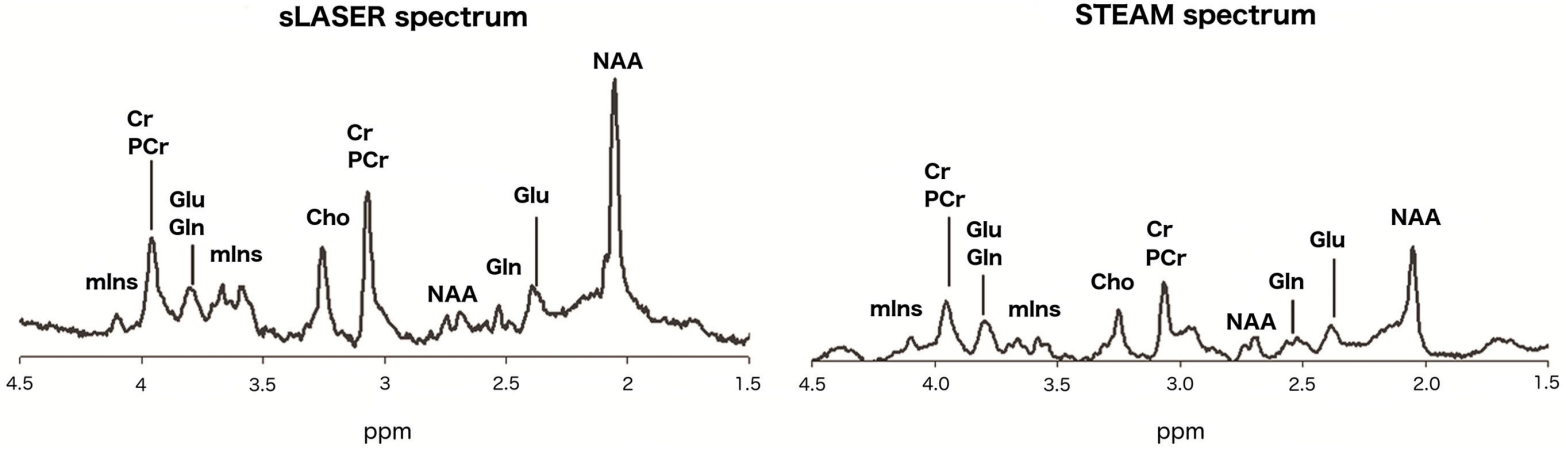
**Typical spectra for the semi-localized by adiabatic selective refocusing (sLASER) sequence (left) and stimulated echo acquisition mode (STEAM) sequence (right)**.

*In vivo* ^1^H magnetic resonance spectroscopy (^1^H-MRS) can be used to determine glutamate levels in the human brain. Glutamate is the primary excitatory neurotransmitter in the mammalian central nervous system. Examining glutamate levels is important when studying a variety of neuropsychiatric conditions, including schizophrenia, bipolar disorder, depression, Alzheimer’s dementia, and anxiety disorders (1). Up until now, the majority of studies examining glutamate in psychiatric disorders using ^1^H-MRS were conducted at magnetic field strengths of 4 T or lower. However, measurement of glutamate with ^1^H-MRS is challenging at lower field strengths, due to its spectral overlap with glutamine. A magnetic field strength of 7 T not only results in an increased signal-to-noise ratio (SNR) but also in an increased chemical shift dispersion. Therefore, the glutamate and glutamine resonances can be adequately separated, and glutamate can be accurately determined (2). The full in-phase glutamate signal can be acquired using short echo time (TE) ^1^H-MRS sequences. For human brain applications, the stimulated echo acquisition mode (STEAM) sequence is the most commonly used method at 7 T for localization For human brain applications, the stimulated echo acquisition mode (STEAM) sequence is the most commonly used method at 7 T for localization (3), in particular for metabolites with a short T_2_ (e.g., glutamate). However, STEAM suffers from severe SNR loss because only half of the available signal can be obtained (Figure 1). Recently, a semi-localized by adiabatic selective refocusing (sLASER) sequence has been developed for application at a magnetic field strength of 7 T. sLASER can be applied to the human brain with a conventional volume head coil, and the full signal can be acquired at short TE with a small chemical shift displacement artifact. The two transmit channels in a conventional volume head coil are driven independently, generating a maximized B_1_^+^ field and allowing the use of short adiabatic refocusing pulses for single-voxel MRS in most of the brain (4).

When performing clinical ^1^H-MRS studies, it is necessary to reliably detect changes in metabolite levels caused by diseases. As mentioned above, glutamate assessment in the human brain has been challenging at low magnetic field strengths due to poor spectral resolution and SNR loss. With the introduction of the sLASER sequence at 7 T, these issues could be overcome. To determine if sLASER can be used as a standard in ^1^H-MRS, studies of glutamate levels in psychiatric disorders, it is needed to assess the reproducibility of glutamate measurement with the sLASER sequence. To evaluate this, experiments were performed at two time-points in eight healthy, age-matched volunteers. Both the STEAM and sLASER sequence were used to measure metabolite concentrations in the frontal as well as the occipital brain region. In addition, we fitted the data into metabolite concentrations using three models, each including a different amount of metabolite basis sets, to evaluate the robustness of the results.

## Materials and Methods

### Subjects

Eight healthy subjects (21–29 years, mean ± SD = 23.9 ± 2.4 years, three males, five females) were scanned twice, with 2 weeks between the measurements. Written informed consent, as approved by the institutional ethics board, was given by all volunteers prior to the examinations. Participants had no major psychiatric or neurological history and no history of drug or alcohol abuse, as tested with the Mini International Neuropsychiatric Interview Plus (MINI-Plus) (5). Participants had no first degree relatives with psychiatric or neurological disorders. We excluded the first measurement with the sLASER sequence in the occipital lobe in one subject because of low spectral quality.

### MR Acquisition

**Figure 2:**
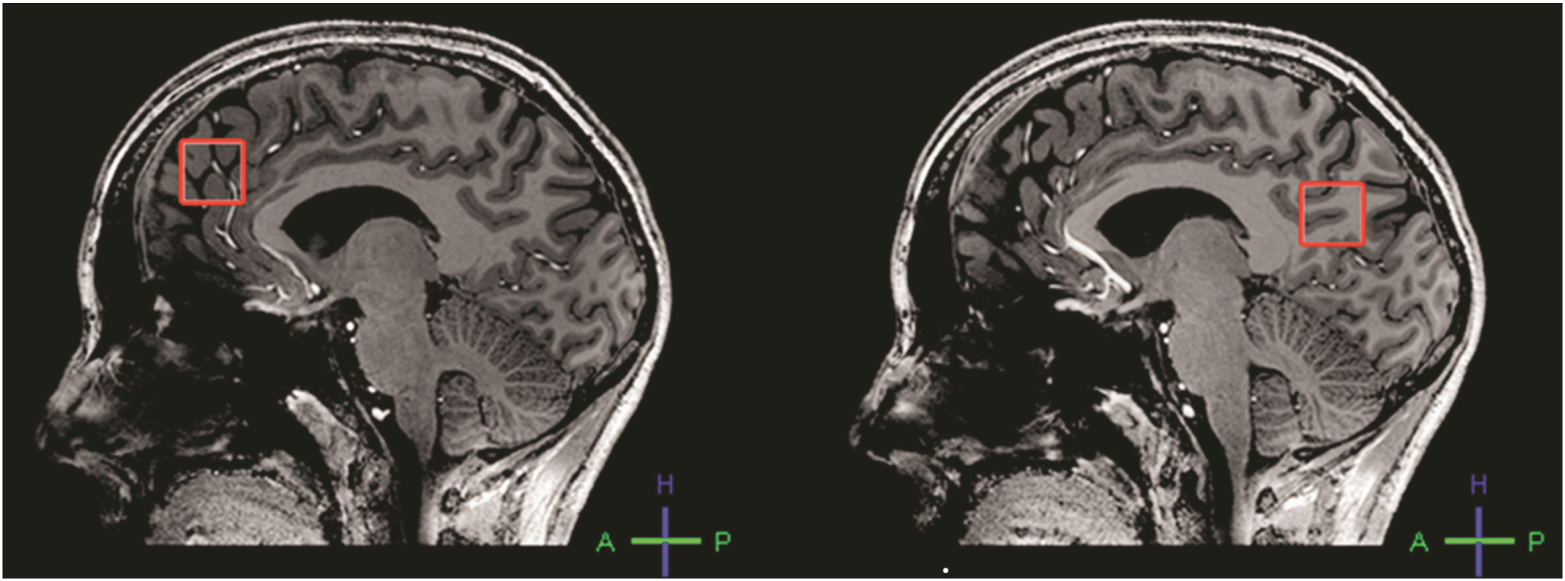
**Placement of the frontal (left) and occipital (right) voxel**.

All investigations were performed on a 7 T whole body MR scanner (Philips, Cleveland, OH, USA). A birdcage transmit head coil was used in dual transmit driven by 2 × 4 kW amplifiers, in combination with a 16-channel receive coil (both Nova Medical, Inc., Burlington, MA, USA). A T_1_-weighted MP-RAGE sequence (450 slices, slice thickness = 0.8 mm, TR = 7 ms, TE = 3 ms, flip angle = 8°, FOV = 250 mm × 200 mm × 180 mm, 312 × 312 acquisition matrix, SENSE factor 2.7, scan duration = 408 s) was obtained for anatomical reference and gray and white matter (GM and WM) tissue classification. ^1^H-MRS experiments were conducted with two short TE sequences, i.e., STEAM (stimulated echo acquisition mode; TE = 7.8 ms, 128 averages, TR = 2 s) and sLASER (semi-localized by adiabatic selective refocusing; TE = 28 ms, 16 averages, TR = 5 s). Voxels (2 cm × 2 cm × 2 cm) were located in the left frontal and left occipital lobe (Figure 2). Non-water suppressed spectra were obtained for quantification (carrier frequency was set to the chemical shift of H_2_O, acquisition time = 10 s). Prior to the MRS exams, second order B_0_ shimming was applied using the FASTERMAP algorithm at the voxel of interest (6, 7). Second, at this location, a high B_1_ field was generated to minimize chemical shift displacement artifacts (8). The highest possible B_1_ field was generated by optimizing the phase of both transmit channels to locally assure constructive B_1_ interferences (4).

### Spectral Fitting and Quantification

Retrospective phase and frequency alignment was performed on all data sets of each measurement (9). Spectral fitting was performed with LCModel-based software implemented in Matlab (10), which uses *a priori* knowledge of the spectral components to fit metabolite resonances (11). Basis sets were generated for the STEAM and sLASER sequence. Three separate fitting procedures were performed on all data sets to examine if the amount of metabolites included in the model influences the reproducibility of the data. In the separate fitting procedures 8 (PC, PE, PCr, NAA, Glu, Gln, GSH, mIns), 12 (Cho, PC, GPC, PE, Cr, PCr, NAA, NAAG, Glu, Gln, GSH, mIns) or 16 (Ace, Asp, Cho, PC, GPC, PE, Cr, PCr, NAA, NAAG, GABA, Glu, Gln, GSH, mIns, Tau) metabolites and a measured macromolecular baseline (12) were fitted to the spectra (Table 1). Metabolite levels were estimated using the water signal as an internal reference and calculated as follows:

$$[\text{met}]=\left(\frac{\frac{{\text{signal}}_{\text{met}}}{{\text{signal}}_{\text{water}}}*\left(\begin{array}{l} \text{volGM}*\left[{\text{water}}_{\text{GM}}\right]\\ \;+\text{volWM}*\left[{\text{water}}_{\text{WM}}\right]\\ \;+\text{volCSF}*\left[{\text{water}}_{\text{pure}}\right] \end{array}\right)}{\text{volGM}+\text{volWM}}\right)$$

where [met] is the metabolite concentration, signal_met_ is the fitted signal intensity of the metabolite, accounting for the number of protons, and signal_water_ is the fitted signal intensity of water, accounting for the number of protons; volGM, volWM, and volCSF are, respectively, the GM content, WM content, and cerebrospinal fluid (CSF) content in the voxel; and [water_GM_], [water_WM_], and [water_pure_] are, respectively, the water concentration in GM, WM, or CSF. For determining the contribution of GM, WM, and CSF of each voxel, the software package SPM8 was used to segment the T_1_-weighted image. In the T_1_-weighted image, the position of the ^1^H-MRS voxel was determined, after which the amount of GM, WM, and CSF in the ^1^H-MRS voxel was computed. To account for differences in transverse relaxation between water and metabolites, a correction was applied based on reported T_2_ values at 7 T of 47 ms on average for water and 107 ms assumed for the metabolites (13).

**Table 1:** **Metabolites that were fitted to the spectra using three different fitting procedures**.

Metabolites	8-metabolite fit	12-metabolite fit	16-metabolite fit
Acetate (Ace)			x
Aspartate (Asp)			x
Choline (Cho)		x	x
Phosphorylcholine (PC)	x	x	x
Glycerophosphorylcholine (GPC)		x	x
Phosphorylethanolamine (PE)	x	x	x
Creatine (Cr)		x	x
Phosphocreatine (PCr)	x	x	x
N-acetyl aspartate (NAA)	x	x	x
N-acetyl aspartyl glutamate (NAAG)		x	x
Gamma-aminobutyric acid (GABA)			x
Glutamate (Glu)	x	x	x
Glutamine (Gln)	x	x	x
Glutathione (GSH)	x	x	x
Myo-inositol (mIns)	x	x	x
Taurine (Tau)			x
Macromolecules (MM)	x	x	x

### Statistical Analysis

To assess the reproducibility for glutamate, and the major spectral components total *N*-acetyl aspartate (NAA) (NAA + NAAG), total creatine (Cr + PCr), and total choline (Cho + PCH + GPC + PE), a test–retest reliability test was performed (SPSS 15.0, Chicago, IL, USA) for each sequence and VOI, by calculating the intraclass correlation coefficient using a two-way mixed model ANOVA. We reported the average measures intraclass correlations (ICC), since it takes into account the average of the values of the two scan sessions. A negative ICC indicates that the measurement is not reliable.

## Results

**Figure 3:**
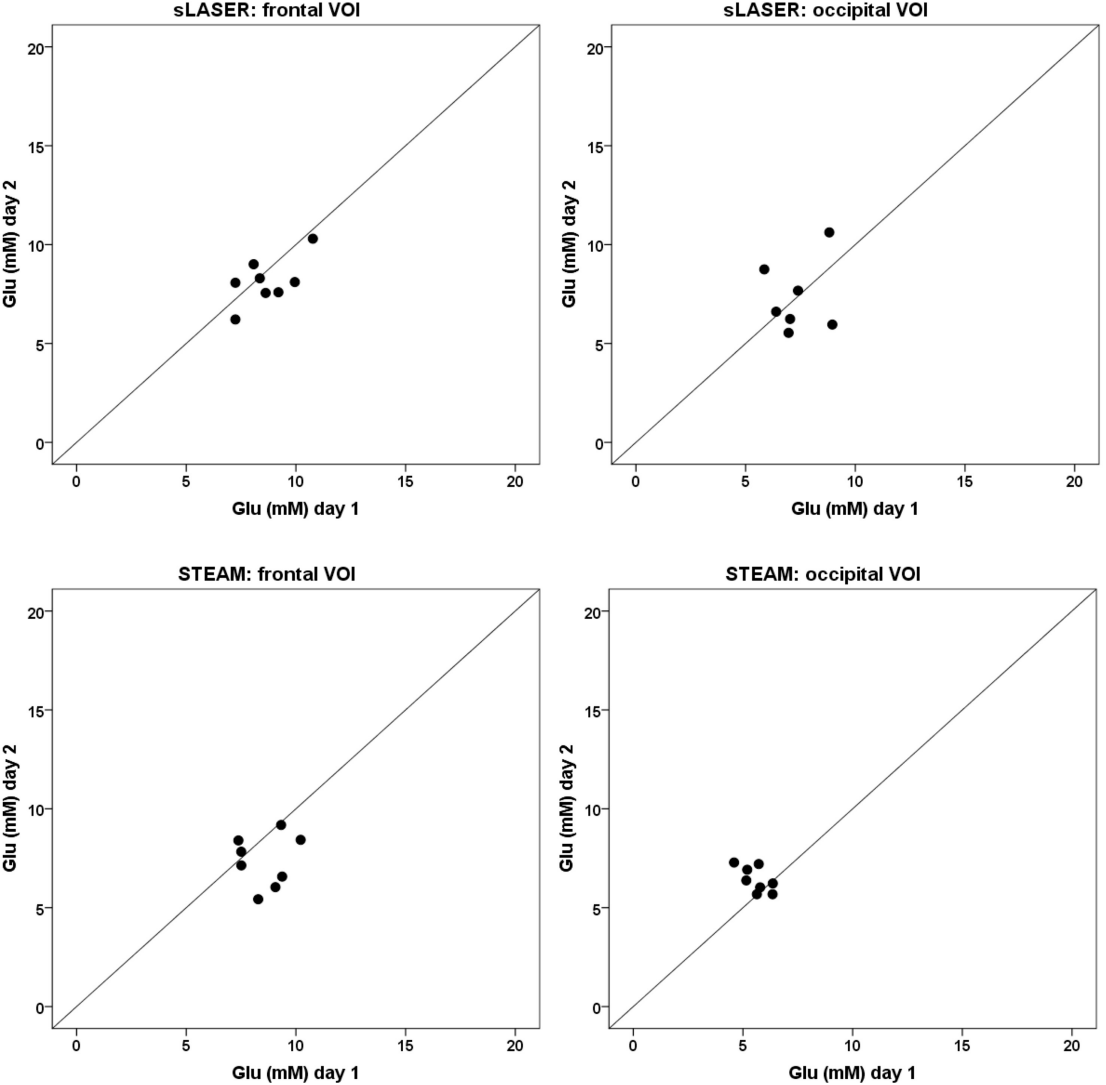
**Glutamate concentrations calculated with an 8-metabolite fit at day 1 (*x*-axis) and day 2 (*y*-axis)**. The line *x* = *y* represents a correlation of +1 between the two measurements. A point-spread along the line *x* = *y* represents detection of mainly physiological variation, a point-spread perpendicular to the line *x* = *y* represents detection of mainly methodological variation.

**Figure 4:**
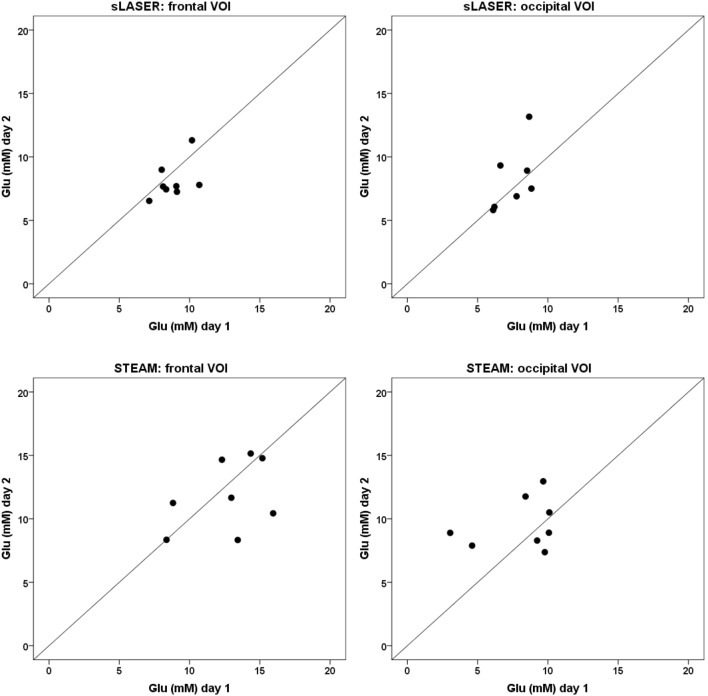
**Glutamate concentrations calculated with a 12-metabolite fit at day 1 (*x*-axis) and day 2 (*y*-axis)**. The line *x* = *y* represents a correlation of +1 between the two measurements. A point-spread along the line *x* = *y* represents detection of mainly physiological variation, a point-spread perpendicular to the line *x* = *y* represents detection of mainly methodological variation.

**Figure 5:**
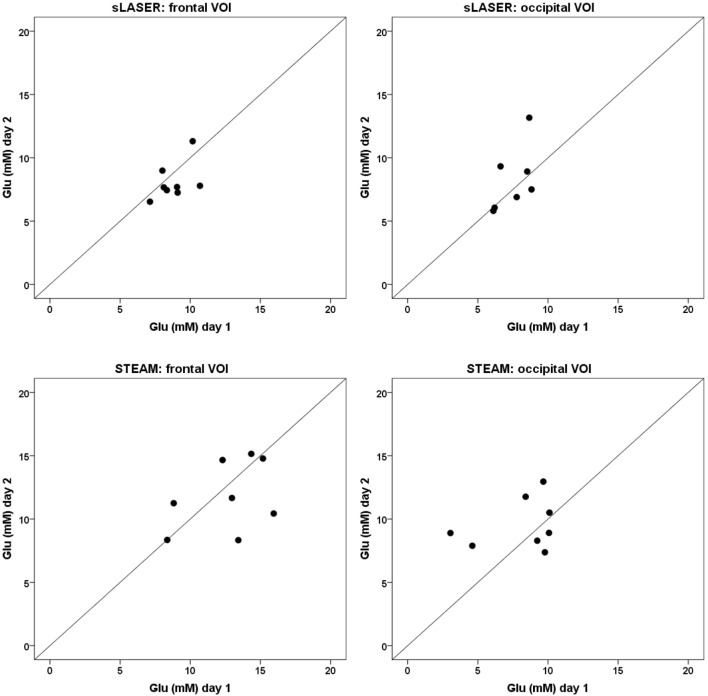
**Glutamate concentrations calculated with a 16-metabolite fit at day 1 (*x*-axis) and day 2 (*y*-axis)**. The line *x* = *y* represents a correlation of +1 between the two measurements. A point-spread along the line *x* = *y* represents detection of mainly physiological variation, a point-spread perpendicular to the line *x* = *y* represents detection of mainly methodological variation.

Glutamate concentrations for all subjects at the two scan sessions and as determined by the three different fitting procedures are shown in Figures 3–5. ICC’s and *p*-values are shown in Table 2. Coefficients of variation values are provided in Table S7 in Supplementary Material. Average metabolite concentrations are shown in Table 3. In the frontal lobe, the sLASER provides significant ICC’s when fitting with 8 or 16-metabolite basis sets.

**Table 2:** **ICC’s and *p*-values for measurement of glutamate concentrations, using semi-localized by adiabatic selective refocusing (sLASER) and stimulated echo acquisition mode (STEAM) in a frontal and occipital VOI, for three different fitting procedures**.

	sLASER				STEAM			
	Frontal		Occipital		Frontal		Occipital	
	Intraclass correlations (ICC)	*p*-Value	ICC	*p*-Value	ICC	*p*-Value	ICC	*p*-Value
8-metabolite fit	0.77	0.04	0.28	0.35	0.17	0.41	−3.62	0.97
12-metabolite fit	0.65	0.09	0.61	0.14	0.58	0.14	0.42	0.24
16-metabolite fit	0.80	0.03	0.34	0.31	0.56	0.15	0.54	0.17

**Table 3:** **Glutamate concentrations (average ± SD, in millimolars) at the first and second measurement, using semi-localized by adiabatic selective refocusing (sLASER) and stimulated echo acquisition mode (STEAM) in a frontal and occipital VOI, for three different fitting procedures**.

	sLASER				STEAM			
	Frontal		Occipital		Frontal		Occipital	
	Day 1	Day 2	Day 1	Day 2	Day 1	Day 2	Day 1	Day 2
8-metabolite fit	8.7 ± 1.2	8.1 ± 1.2	7.3 ± 1.2	7.5 ± 1.8	8.6 ± 1.1	7.4 ± 1.3	5.6 ± 0.6	6.4 ± 0.6
12-metabolite fit	12.6 ± 1.7	11.5 ± 2.1	10.8 ± 1.7	12.1 ± 3.5	18.1 ± 4.0	16.9 ± 4.0	11.6 ± 3.9	13.7 ± 2.8
16-metabolite fit	13.6 ± 2.3	12.5 ± 2.5	11.2 ± 1.9	11.7 ± 2.1	19.8 ± 3.9	19.3 ± 5.1	12.9 ± 6.3	15.9 ± 2.4

To evaluate the precision of the quantification of the most commonly obtained metabolites, average metabolite concentrations, ICC’s, and *p*-values are shown in the supplemental figures and tables (Figures S1–S3 in Supplementary Material and Tables S1 and S2 in Supplementary Material for NAA, Figures S4–S6 in Supplementary Material and Tables S3 and S4 in Supplementary Material for Cr, and Figures S7–S9 in Supplementary Material and Tables S5 and S6 in Supplementary Material for Cho).

For the occipital lobe, a significant ICC was found only for NAA measured with sLASER and fitted with the 12-metabolite basis set. Bland–Altman plots for significant ICC’s are shown in Figure S10 in Supplementary Material.

## Discussion

In this study, we estimated the reproducibility of glutamate measurements using the STEAM and sLASER sequence at 7 T in two different areas of the human brain. As compared to the commonly used STEAM sequence, sLASER seems to be a more robust method for determining glutamate levels. It produces similar results at different time-points and is sensitive enough to detect physiological differences between subjects. Particularly, in the frontal brain region that plays an important role in psychiatric disorders (14–18), glutamate concentrations measured with sLASER show a high reproducibility.

Concentrations of NAA, creatine, and choline are expected to remain stable over subjects, particularly in the small age range used in this study (19, 20), hence one would expect lower ICC’s for these metabolites, as the between subjects variance is low. The results for sLASER indeed show that the ICC’s for NAA, creatine, and choline are lower than the ICC computed for glutamate. This has already been shown for sLASER at 3 T (21). In contrast to glutamate, NAA, creatine, and choline do not suffer from overlap with other metabolites at lower field strength and show low between subjects variances. Therefore, measurements of these metabolites at 7 T only result in a higher SNR, while the low ICC’s remain.

We note that, in contrast to the sLASER measurements, a clear difference in variance can be observed between the two measurements using STEAM (see for instance Figure 3). This suggests that the STEAM sequence is more sensitive than the sLASER sequence to external factors that are apparently difficult to control as we tried to keep the conditions in our experiments the same as much as possible. As mentioned before, STEAM suffers from reduced localization accuracy as compared to sLASER, which may compromise the precision of the measurement. Also, the longer measurements times used with the STEAM sequence to partly compensate SNR loss, may have caused reduced stability. Additionally, a fitting procedure including 12 or more metabolite basis sets seems to more robustly display measured metabolite levels. It is known that omitting basis sets from metabolites that are indeed present in the tissue of interest leads to a systematic bias and potential overlap of the fitted metabolite resonances and thereby detects physiological and methodological variations less accurately (22, 23). This seems to be the case in a fitting procedure that includes basis sets for only eight metabolites, for which we generally observed small within and between subjects variations, whereas fitting procedures including basis sets for 12 or 16 metabolites show larger variations within and between subjects.

Several limitations have to be considered when interpreting the results of this study. First, only eight subjects were examined. Although this was not enough to establish the reliability for the STEAM sequence, it was enough to establish the reliability of the sLASER sequence. This finding speaks in favor of the sLASER sequence. On the other hand, application of the STEAM sequence in clinical studies is easier since it does not require additional hardware modifications. To reach a short TE on the system we used, the sLASER required a dual transmit option. This may not be routinely available on all 7-T MR systems, and it also requires slightly more scan preparation, e.g., determination of the optimal phase of the two input channels. If fully automated, however, this would only require several seconds.

A potential cause of variation between repeated measurements might be the manual positioning of the VOIs. Differences in location may result in different contributions of GM, WM, and CSF, which may affect the measured metabolite levels (21). However, the ICC’s of GM and WM content between the first and second measurement in the frontal (GM: ICC = 0.89, *p* < 0.01; WM: ICC = 0.87, *p* < 0.01) and the occipital voxel (GM: ICC = 0.69, *p* = 0.07; WM: ICC = 0.67, *p* = 0.09) indicate only small variations in positioning. Larger variations in positioning and thus slightly smaller ICC’s for GM and WM content, could explain the fact that no significant ICC’s for metabolite levels were found in the occipital region. Also, we corrected the measured metabolite levels for contribution of GM, WM, and CSF by tissue segmentation of the VOIs based on the T_1_-weighted image. While this corrects for the differences in water content and assumed absence of glutamate in CSF, it does not correct for differences in glutamate between GM and WM. However, it is not plausible that systematic variations in GM and WM content of the VOIs are causing high ICC’s for glutamate concentrations, since we did not find significant correlations between glutamate concentrations and GM content, and including GM or WM fractions as a regressor in the analyses did not yield different results. *For completeness, the analysis was repeated on the uncorrected data (no correction for GM and WM fractions), but this did not change the nature of our findings*.

We conclude that the sLASER sequence can be successfully applied for glutamate measurements in the human brain at 7 T. MR spectra acquired at different time-points, and in a well-controlled population, are comparable and robust and the variance is mainly caused by physiological differences between subjects, since the methodological variation is reduced when using sLASER compared to STEAM. This is particularly beneficial when studying populations that are difficult to recruit, since sLASER requires a smaller sample size than STEAM to detect physiological differences between subjects.

## Ethics Statement

This study was carried out in accordance with the recommendations of the institutional ethics board (METC) with written informed consent from all subjects. All subjects gave written informed consent in accordance with the Declaration of Helsinki. The protocol was approved by the institutional ethics board (METC).

## Author Contributions

AM: involved in designing the experiment, acquired the data, performed the analysis of the results, and wrote the initial version of the manuscript. VB: involved in data acquisition and revising the manuscript. PL: involved in revising the manuscript. HH, DK, and RM: involved in designing the experiment, analysis of the results, and revising the manuscript.

## Conflict of Interest Statement

The authors declare that the research was conducted in the absence of any commercial or financial relationships that could be construed as a potential conflict of interest.
