# Supplementary material for: Detection of Glutamate Alterations in the Human Brain Using 1H-MRS: Comparison of STEAM and sLASER at 7 T
Source: Front Psychiatry. 2017 Apr 21;8:60. doi: 10.3389/fpsyt.2017.00060 (PMC5399075; doi:10.3389/fpsyt.2017.00060)
Supplement: Supplementary file 1 [file data_sheet_1.docx]

**Supplement**

**Please note that choline and creatine are not part of the 8 metabolite fit model and therefore the reported concentrations for that model are less reliable.**

Supplemental table 1: ICC’s and p-values for measurement of N-acetyl aspartate concentrations, using sLASER and STEAM in a frontal and occipital VOI, for three different fitting procedures.

|  | sLASER | | | | STEAM | | | |
| --- | --- | --- | --- | --- | --- | --- | --- | --- |
|  | frontal | | occipital | | frontal | | occipital | |
|  | ICC | p | ICC | p | ICC | p | ICC | p |
| 8 metabolite fit | 0.11 | 0.44 | 0.23 | 0.38 | -0.45 | 0.68 | -1.85 | 0.91 |
| 12 metabolite fit | 0.50 | 0.19 | 0.81 | 0.03 | 0.06 | 0.47 | -1.12 | 0.83 |
| 16 metabolite fit | 0.54 | 0.17 | 0.58 | 0.16 | -0.31 | 0.64 | -1.15 | 0.83 |

Supplemental table 2: N-acetyl aspartate concentrations (average ± SD, in mM) at the first and second measurement, using sLASER and STEAM in a frontal and occipital VOI, for three different fitting procedures.

|  | sLASER | | | | STEAM | | | |
| --- | --- | --- | --- | --- | --- | --- | --- | --- |
|  | frontal | | Occipital | | frontal | | occipital | |
|  | Day 1 | Day 2 | Day 1 | Day 2 | Day 1 | Day 2 | Day 1 | Day 2 |
| 8 metabolite fit | 10.8±2.1 | 9.9±0.8 | 10.7±1.6 | 11.2±1.0 | 8.0±2.5 | 6.5±0.9 | 6.3±0.6 | 6.8±1.2 |
| 12 metabolite fit | 10.9±1.7 | 9.8±0.8 | 10.9±1.4 | 11.3±1.4 | 16.6±2.5 | 15.5±1.4 | 14.7±1.2 | 16.6±3.3 |
| 16 metabolite fit | 10.7±1.7 | 9.7±0.8 | 10.8±1.4 | 11.2±0.9 | 16.7±3.1 | 15.7±1.8 | 14.8±1.5 | 16.7±3.1 |


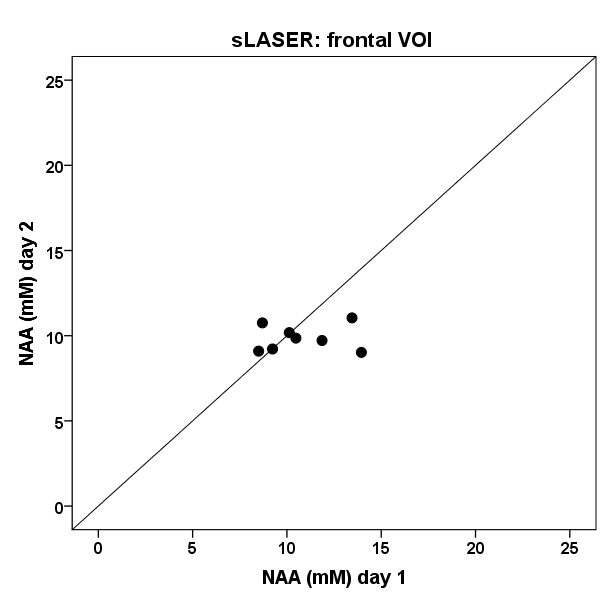

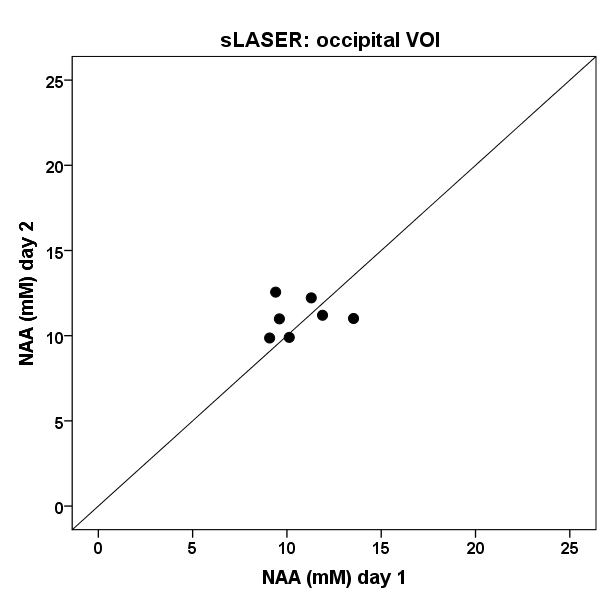

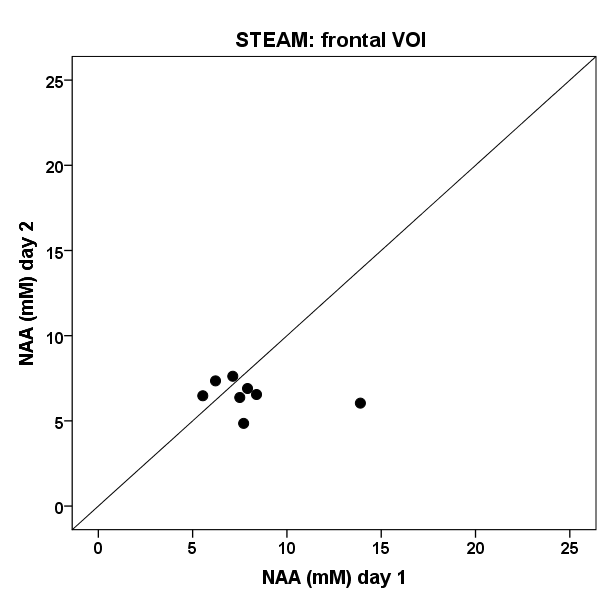

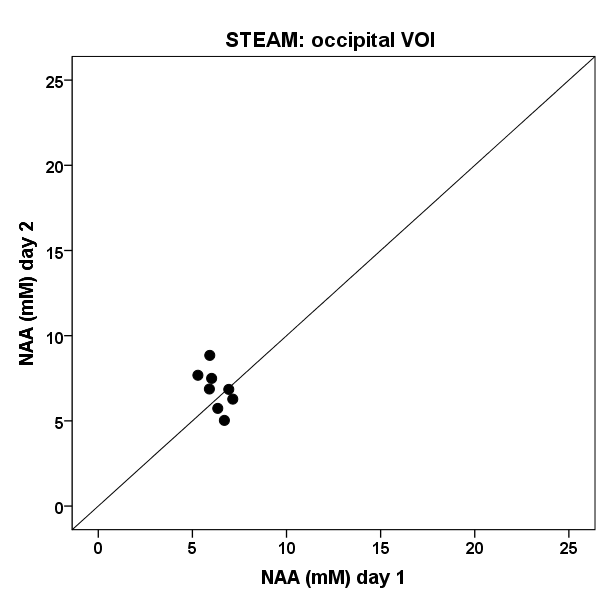


Supplemental Figure 1: NAA concentrations calculated with an 8 metabolite fit at day 1 (x-axis) and day 2 (y-axis). The line x=y represents a correlation of +1 between the two measurements. A point-spread along the line x=y represents detection of mainly physiological variation, a point-spread perpendicular to the line x=y represents detection of mainly methodological variation.


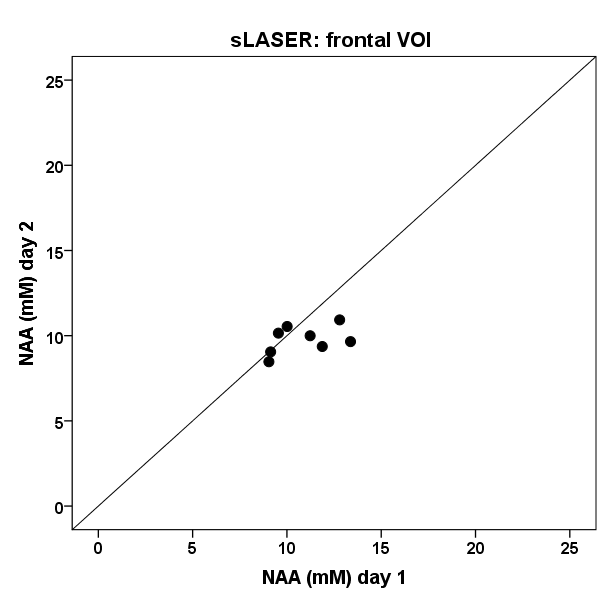

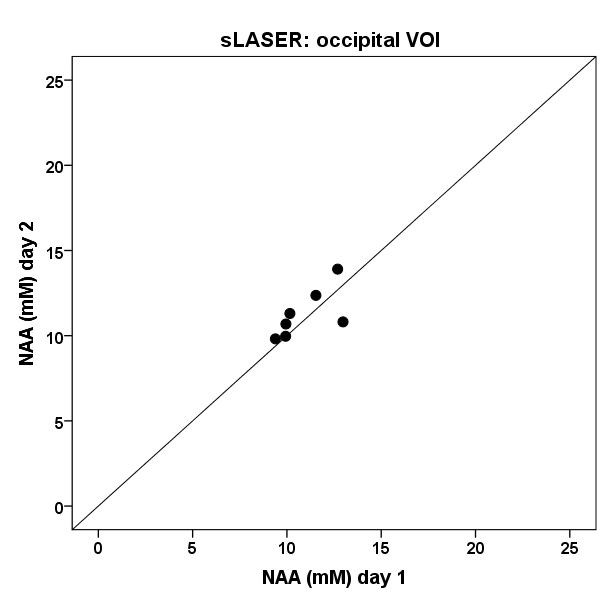

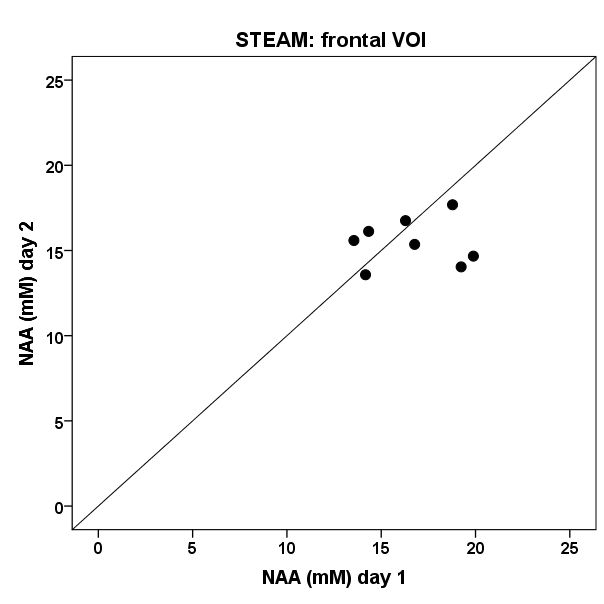

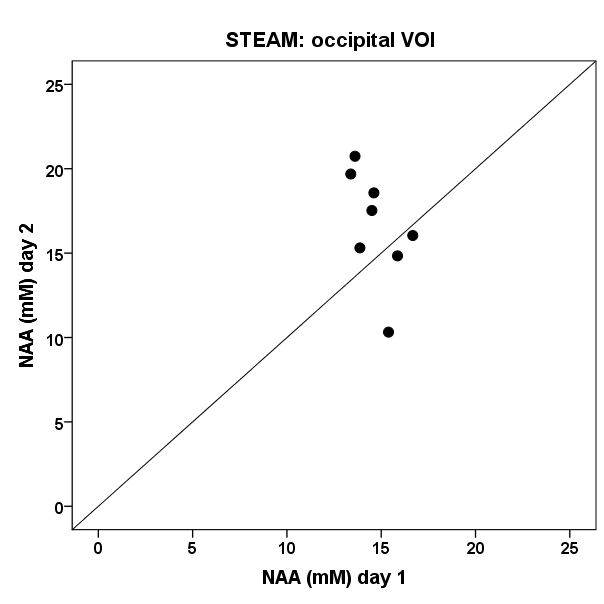


Supplemental figure 2: NAA concentrations calculated with a 12 metabolite fit at day 1 (x-axis) and day 2 (y-axis). The line x=y represents a correlation of +1 between the two measurements. A point-spread along the line x=y represents detection of mainly physiological variation, a point-spread perpendicular to the line x=y represents detection of mainly methodological variation.


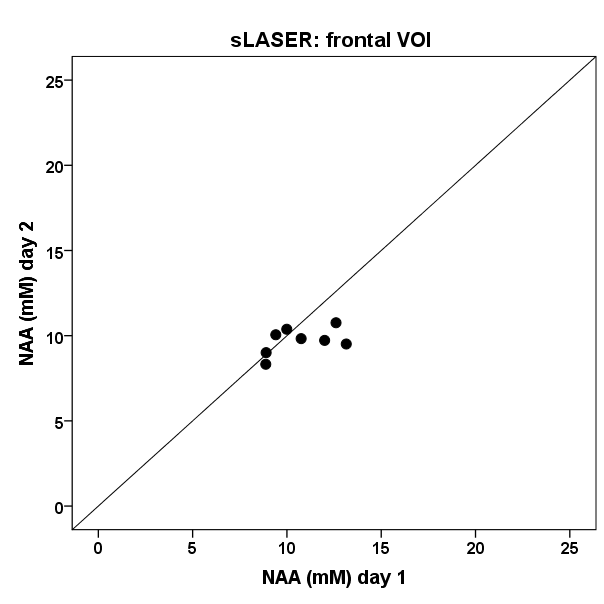

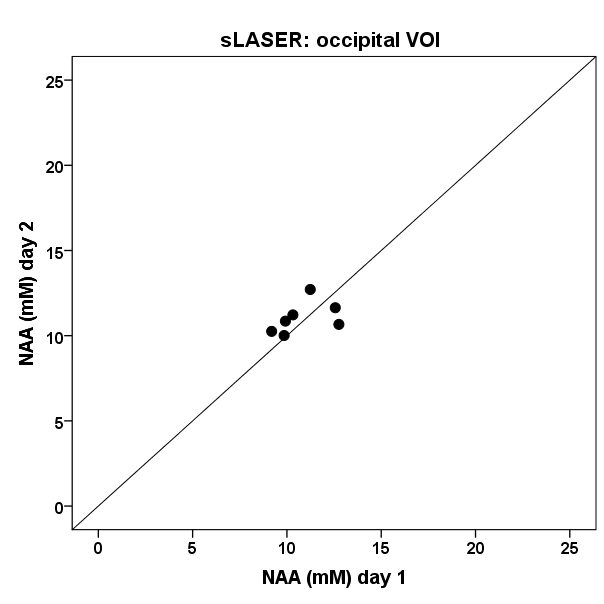

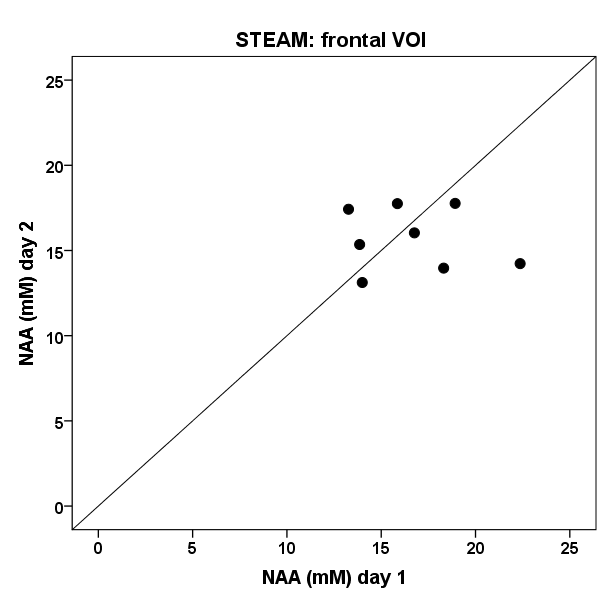

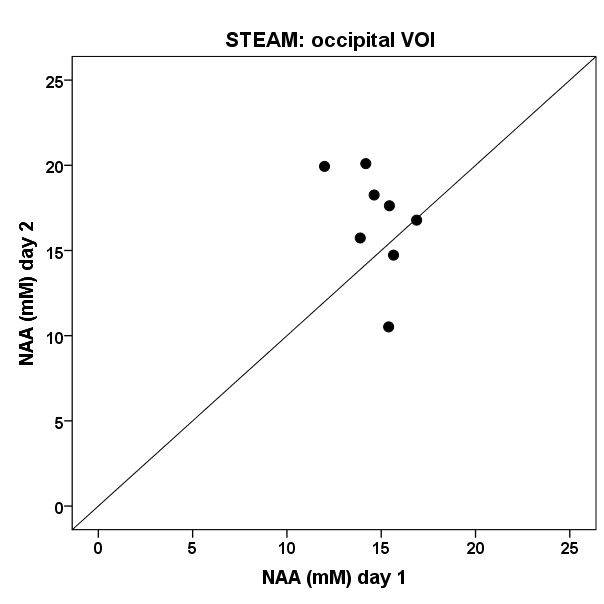


Supplemental figure 3: NAA concentrations calculated with a 16 metabolite fit at day 1 (x-axis) and day 2 (y-axis). The line x=y represents a correlation of +1 between the two measurements. A point-spread along the line x=y represents detection of mainly physiological variation, a point-spread perpendicular to the line x=y represents detection of mainly methodological variation.

Supplemental table 3: ICC’s and p-values for measurement of creatine concentrations, using sLASER and STEAM in a frontal and occipital VOI, for three different fitting procedures.

|  | sLASER | | | | STEAM | | | |
| --- | --- | --- | --- | --- | --- | --- | --- | --- |
|  | frontal | | Occipital | | frontal | | occipital | |
|  | ICC | p | ICC | P | ICC | p | ICC | p |
| 8 metabolite fit | 0.16 | 0.41 | 0.16 | 0.42 | 0.08 | 0.46 | -0.17 | 0.58 |
| 12 metabolite fit | 0.09 | 0.45 | 0.60 | 0.15 | -0.33 | 0.64 | -0.03 | 0.52 |
| 16 metabolite fit | -0.67 | 0.74 | 0.27 | 0.36 | -0.78 | 0.77 | 0.41 | 0.25 |

Supplemental table 4: Creatine concentrations (average ± SD, in mM) at the first and second measurement, using sLASER and STEAM in a frontal and occipital VOI, for three different fitting procedures.

|  | sLASER | | | | STEAM | | | |
| --- | --- | --- | --- | --- | --- | --- | --- | --- |
|  | frontal | | Occipital | | frontal | | occipital | |
|  | Day 1 | Day 2 | Day 1 | Day 2 | Day 1 | Day 2 | Day 1 | Day 2 |
| 8 metabolite fit | 8.0±1.3 | 7.6±0.4 | 6.9±0.9 | 6.9±0.5 | 6.2±1.4 | 5.2±0.3 | 5.0±0.5 | 4.8±1.0 |
| 12 metabolite fit | 7.7±1.1 | 7.2±0.5 | 6.6±0.9 | 7.8±2.1 | 11.2±1.1 | 10.3±0.9 | 10.1±1.7 | 10.7±3.7 |
| 16 metabolite fit | 8.5±1.2 | 8.4±0.6 | 7.1±0.9 | 7.0±0.8 | 13.0±1.2 | 12.7±1.3 | 10.13.1 | 12.5±1.9 |


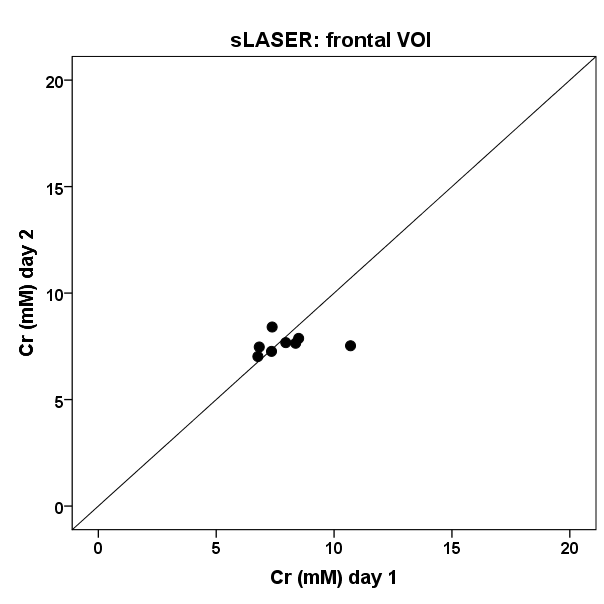

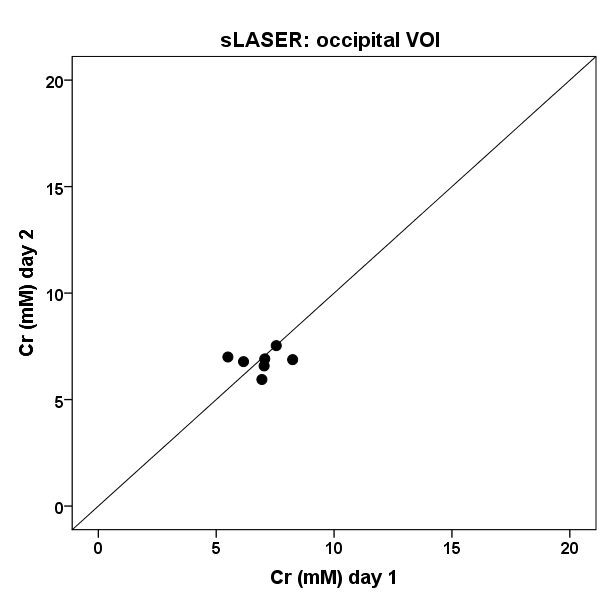

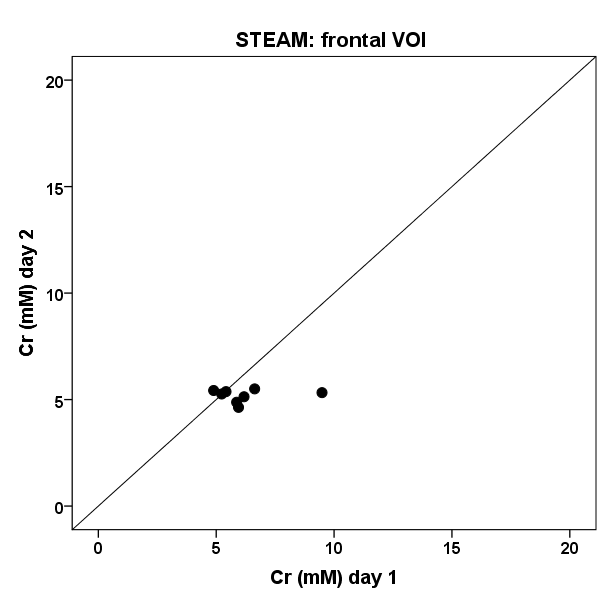

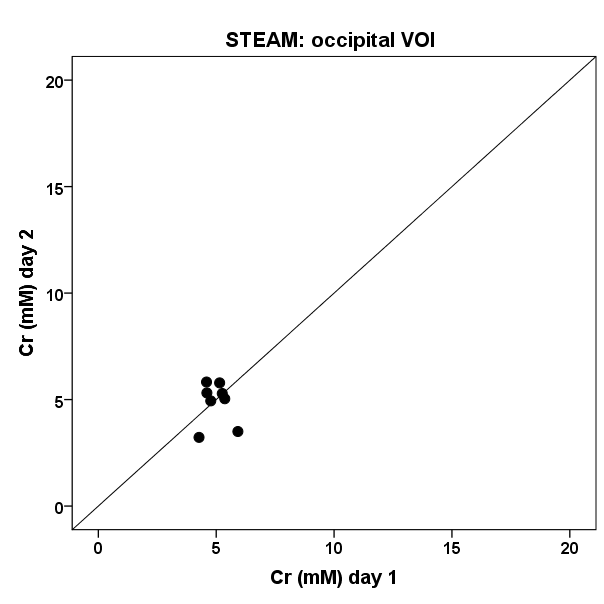


Supplemental figure 4: Creatine concentrations calculated with an 8 metabolite fit at day 1 (x-axis) and day 2 (y-axis). The line x=y represents a correlation of +1 between the two measurements. A point-spread along the line x=y represents detection of mainly physiological variation, a point-spread perpendicular to the line x=y represents detection of mainly methodological variation.


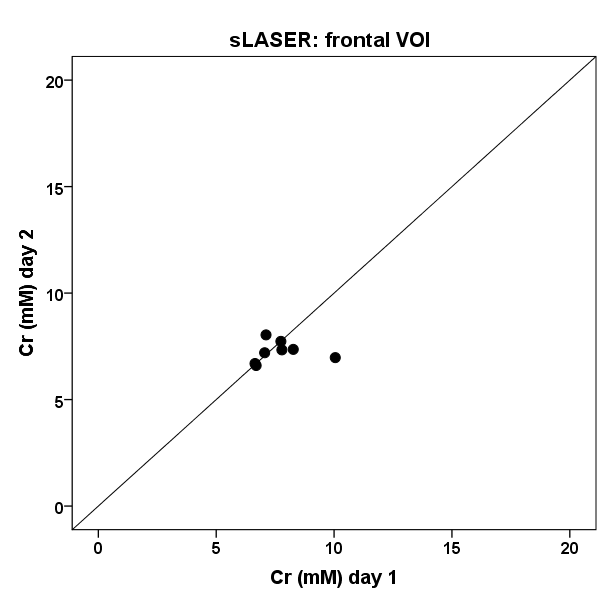

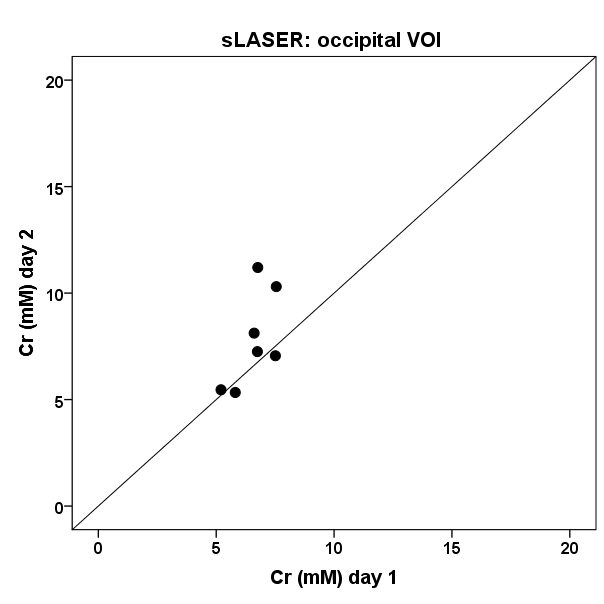

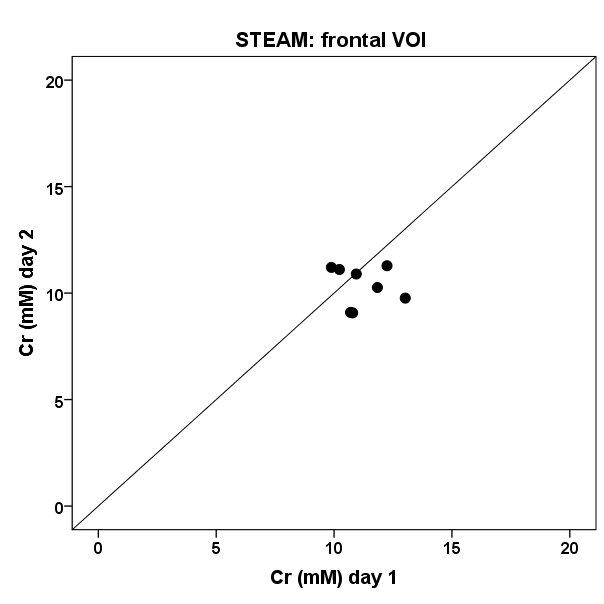

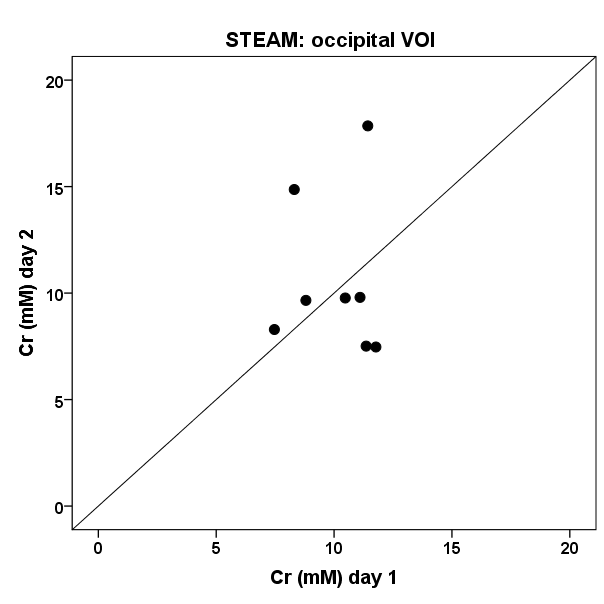


Supplemental figure 5: Creatine concentrations calculated with a 12 metabolite fit at day 1 (x-axis) and day 2 (y-axis). The line x=y represents a correlation of +1 between the two measurements. A point-spread along the line x=y represents detection of mainly physiological variation, a point-spread perpendicular to the line x=y represents detection of mainly methodological variation.


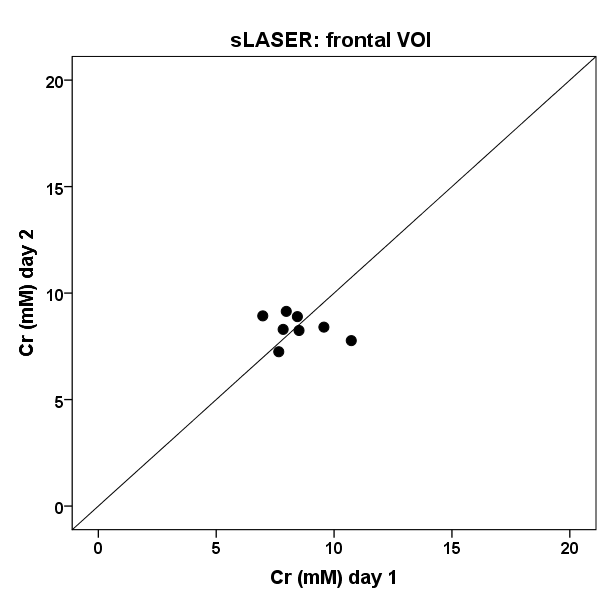

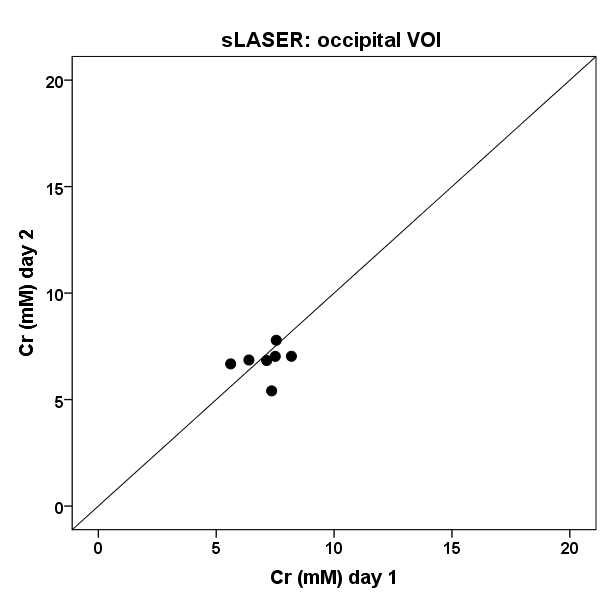

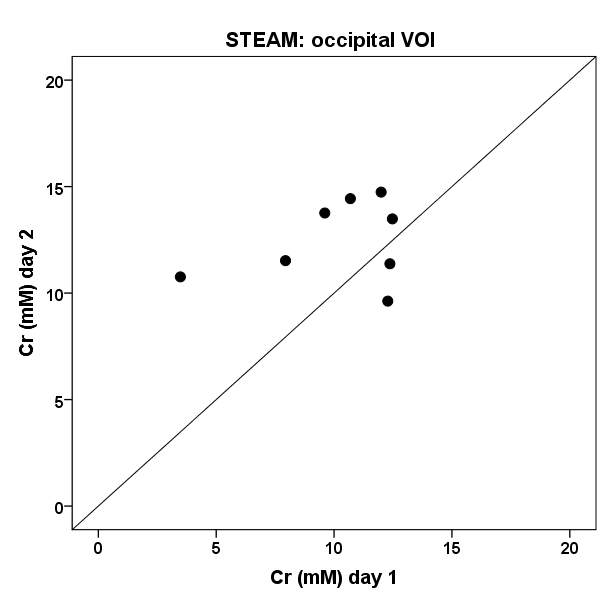

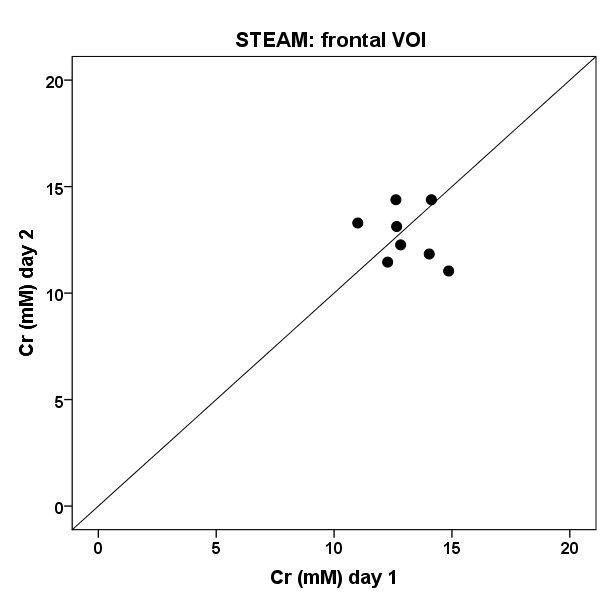


Supplemental figure 6: Creatine concentrations calculated with a 16 metabolite fit at day 1 (x-axis) and day 2 (y-axis). The line x=y represents a correlation of +1 between the two measurements. A point-spread along the line x=y represents detection of mainly physiological variation, a point-spread perpendicular to the line x=y represents detection of mainly methodological variation.

Supplemental table 5: ICC’s and p-values for measurement of choline concentrations, using sLASER and STEAM in a frontal and occipital VOI, for three different fitting procedures.

|  | sLASER | | | | STEAM | | | |
| --- | --- | --- | --- | --- | --- | --- | --- | --- |
|  | frontal | | occipital | | frontal | | occipital | |
|  | ICC | p | ICC | P | ICC | p | ICC | p |
| 8 metabolite fit | 0.08 | 0.46 | 0.61 | 0.14 | 0.22 | 0.38 | 0.54 | 0.16 |
| 12 metabolite fit | 0.39 | 0.27 | 0.74 | 0.06 | 0.36 | 0.28 | -0.01 | 0.51 |
| 16 metabolite fit | -0.31 | 0.64 | 0.58 | 0.16 | -0.72 | 0.76 | 0.12 | 0.43 |

Supplemental table 6: Choline concentrations (average ± SD, in mM) at the first and second measurement, using sLASER and STEAM in a frontal and occipital VOI, for three different fitting procedures.

|  | sLASER | | | | STEAM | | | |
| --- | --- | --- | --- | --- | --- | --- | --- | --- |
|  | frontal | | Occipital | | frontal | | occipital | |
|  | Day 1 | Day 2 | Day 1 | Day 2 | Day 1 | Day 2 | Day 1 | Day 2 |
| 8 metabolite fit | 2.2±0.6 | 2.0±0.8 | 2.1±0.7 | 1.8±0.7 | 2.2±1.3 | 1.2±0.4 | 1.2±0.3 | 1.5±0.8 |
| 12 metabolite fit | 2.0±0.4 | 1.9±0.2 | 1.6±0.3 | 1.7±0.4 | 3.8±1.0 | 3.0±0.4 | 2.0±0.3 | 2.3±0.7 |
| 16 metabolite fit | 2.0±0.4 | 1.9±0.3 | 1.6±0.3 | 1.5±0.1 | 5.0±1.0 | 2.9±0.5 | 3.7±1.5 | 3.5±1.8 |


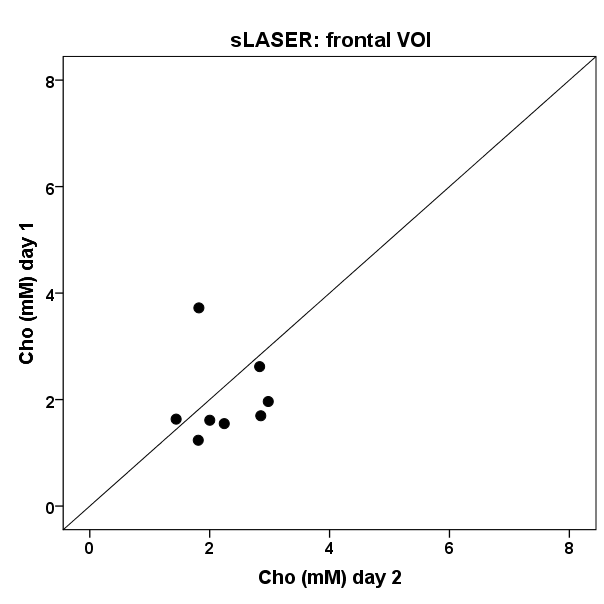

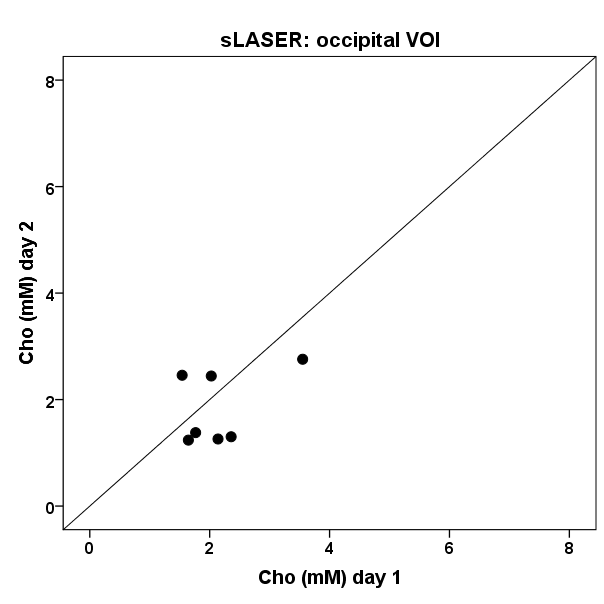

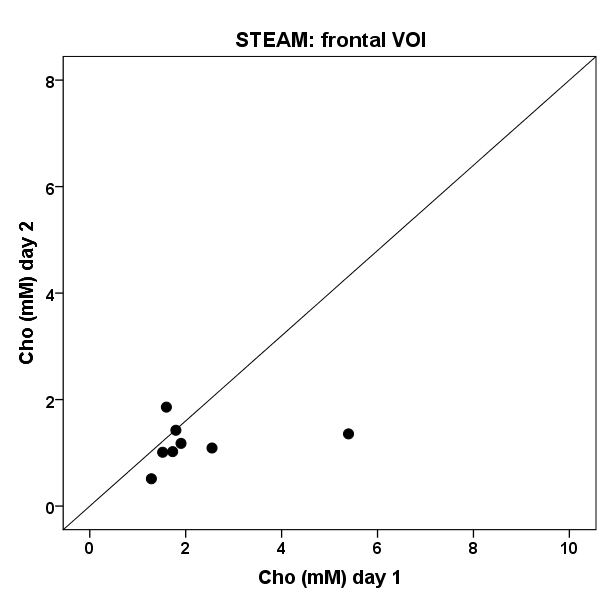

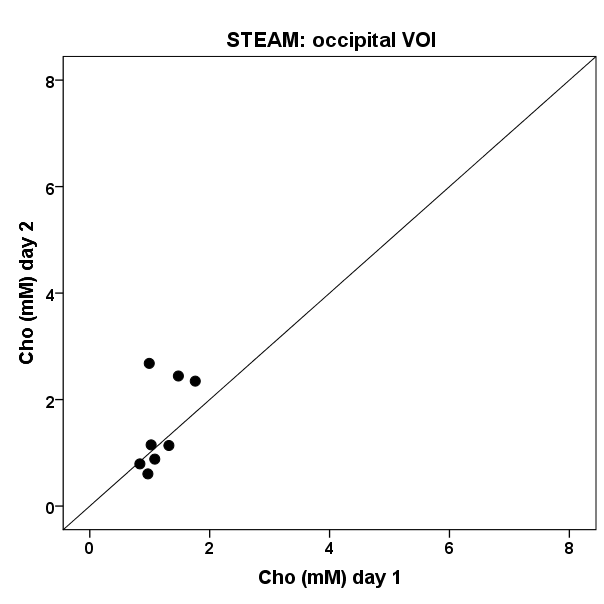


Supplemental figure 7: Choline concentrations calculated with an 8 metabolite fit at day 1 (x-axis) and day 2 (y-axis). The line x=y represents a correlation of +1 between the two measurements. A point-spread along the line x=y represents detection of mainly physiological variation, a point-spread perpendicular to the line x=y represents detection of mainly methodological variation.


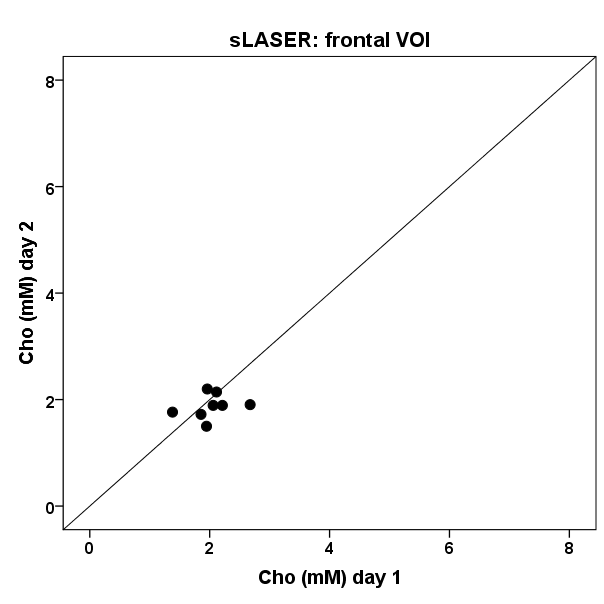

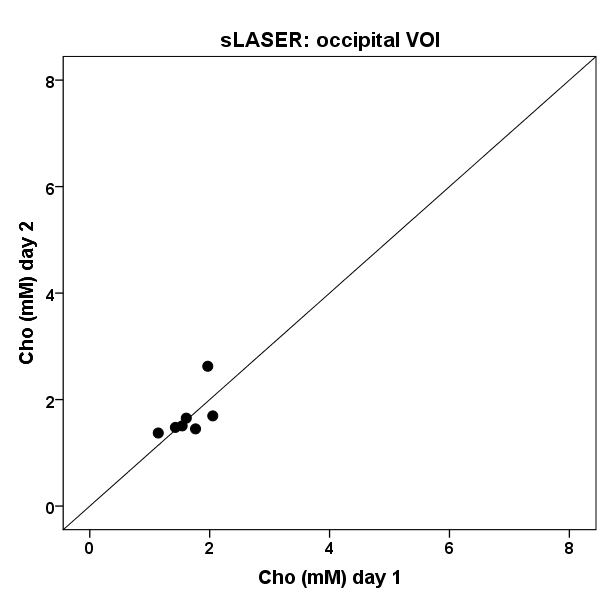

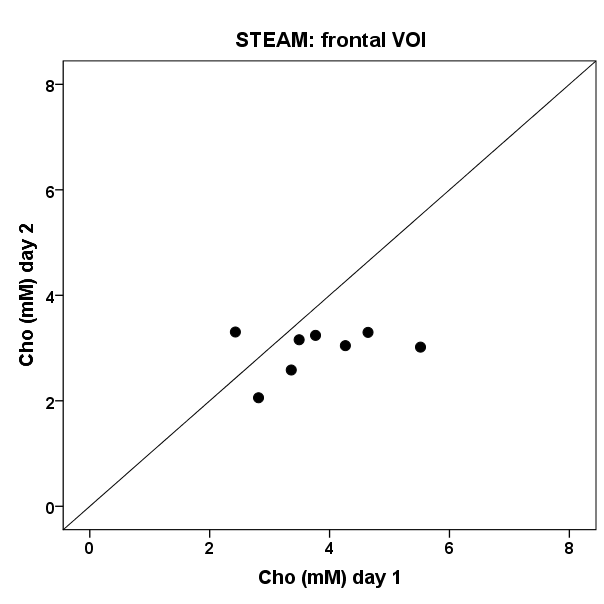

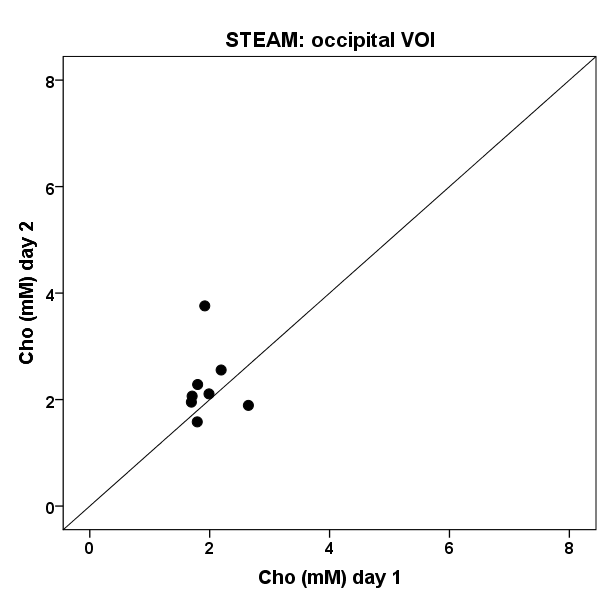


Supplemental figure 8: Choline concentrations calculated with a 12 metabolite fit at day 1 (x-axis) and day 2 (y-axis). The line x=y represents a correlation of +1 between the two measurements. A point-spread along the line x=y represents detection of mainly physiological variation, a point-spread perpendicular to the line x=y represents detection of mainly methodological variation.


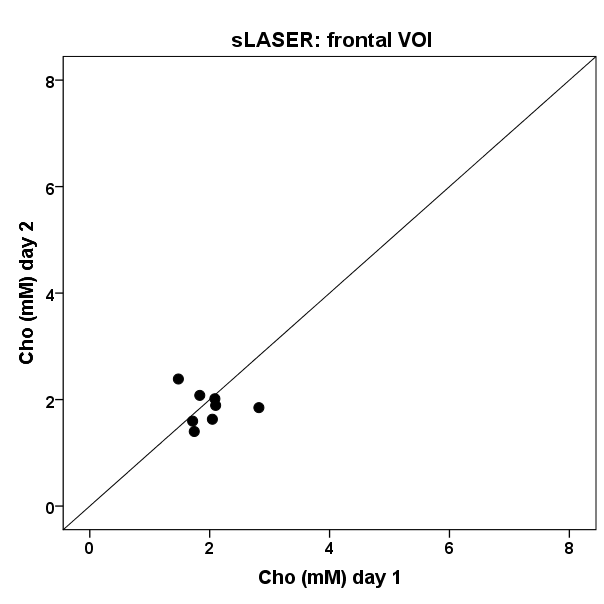

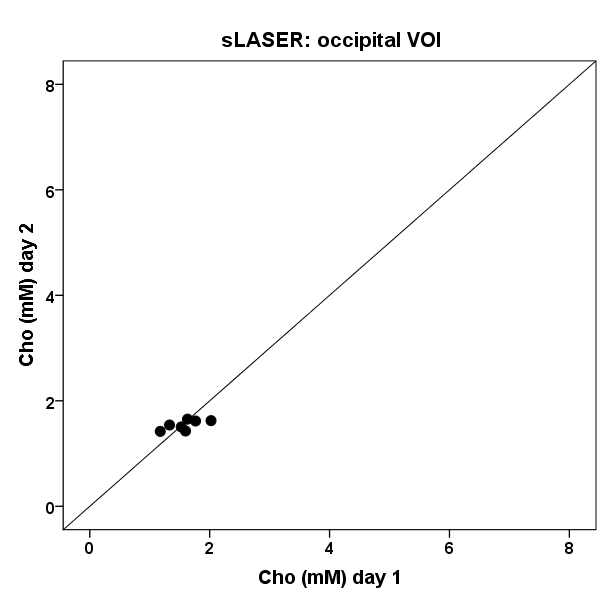

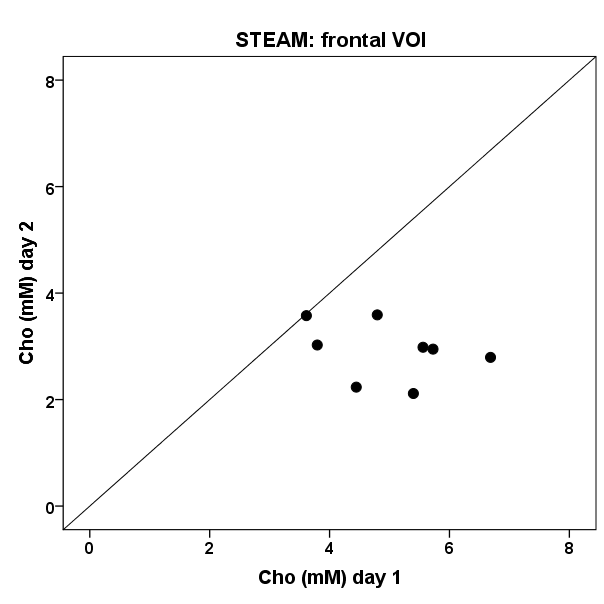

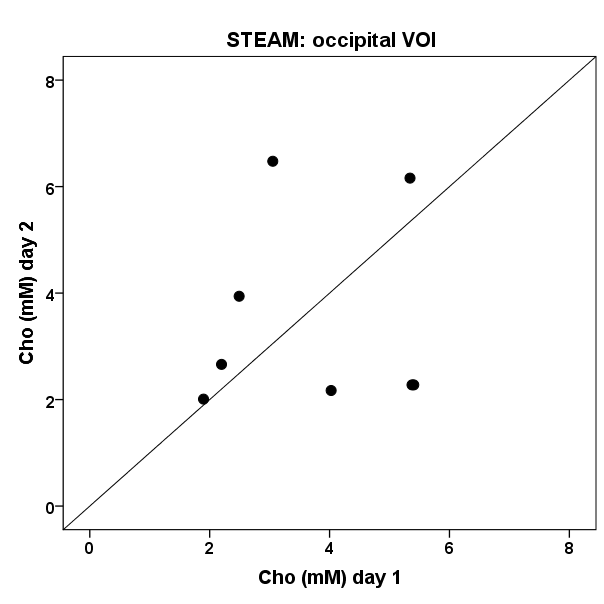


Supplemental figure 9: Choline concentrations calculated with a 16 metabolite fit at day 1 (x-axis) and day 2 (y-axis). The line x=y represents a correlation of +1 between the two measurements. A point-spread along the line x=y represents detection of mainly physiological variation, a point-spread perpendicular to the line x=y represents detection of mainly methodological variation.

**Supplemental table 7: Coefficients of variation.**

| 8 metab fit | sLASER frontal | sLASER occipital* | STEAM frontal | STEAM occipital |
| --- | --- | --- | --- | --- |
| Glu | 12,44% | 26,91% | 20,11% | 18,84% |
| NAA | 20,95% | 16,37% | 40,08% | 24,92% |
| Cr | 16,45% | 14,13% | 25,23% | 23,51% |
| Cho | 45,55% | 36,42% | 76,57% | 52,83% |
|  |  |  |  |  |
| 12 metab fit | sLASER frontal | sLASER occipital* | STEAM frontal | STEAM occipital |
| Glu | 16,04% | 26,82% | 24,80% | 32,64% |
| NAA | 14,78% | 10,52% | 17,40% | 25,95% |
| Cr | 15,98% | 25,28% | 14,04% | 39,68% |
| Cho | 19,10% | 20,66% | 28,46% | 34,68% |
|  |  |  |  |  |
| 16 metab fit | sLASER frontal | sLASER occipital* | STEAM frontal | STEAM occipital |
| Glu | 15,16% | 22,09% | 25,84% | 37,09% |
| NAA | 14,50% | 11,76% | 23,75% | 25,61% |
| Cr | 17,92% | 14,62% | 15,58% | 27,87% |
| Cho | 28,36% | 14,44% | 33,31% | 63,82% |
|  |  |  |  |  |
| CoV=SD(day 2- day 1)/ mean | | | | |
| * here subject #2 is excluded because data for day 2 is missing. | | | | |


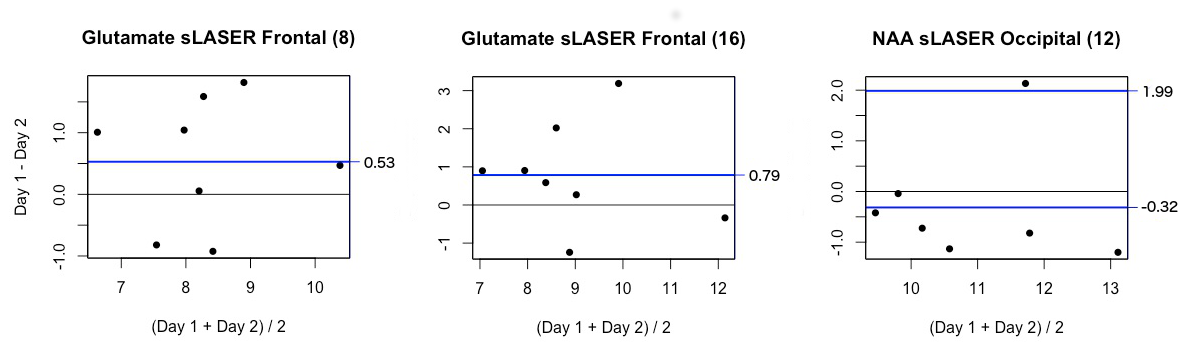


**Supplemental figure 10: Bland-Altman plots for the reported significant correlations.**

**Limits of agreement (difference, 2.5%, 95%, SD(difference) ) for Glutamate sLASER Frontal 8 metabolites, Glutamate sLASER Frontal 16 metabolites and NAA sLASER Occipital 12 metabolites were (0.53, -1.53, 2.59, 1.03), (0.79,-1.95, 3.52, 1.37) and (0.32 ,-2.62, 1.99, 1.15), respectively.**
